# Cardiometabolic and Anthropometric Outcomes of Intermittent Fasting Among Civil Servants With Overweight and Obesity: Study Protocol for a Nonrandomized Controlled Trial

**DOI:** 10.2196/33801

**Published:** 2022-08-05

**Authors:** Shazana Rifham Abdullah, Nur Hayati Azizul, Ruziana Mona Wan Mohd Zin, Nur Suffia Sulaiman, Norhayati Mustafa Khalid, Roshan Jahn Mohd Salim Mullahi Jahn, Muhamad Khairul Nazrin Khalil, Norhashimah Abu Seman, Nur Azlin Zainal Abidin, Azizan Ali, You Zhuan Tan, Azahadi Omar, Mohammad Zabri Johari, Nur Shahida Abdul Aziz, Azli Baharudin, Zamtira Seman, Norazizah Ibrahim Wong, Mona Lisa Md Rasip, Hayati Mohd Yusof, Mohd Fairulnizal Md Noh

**Affiliations:** 1 Nutrition, Metabolism and Cardiovascular Research Centre, Institute for Medical Research, National Institutes of Health Ministry of Health Shah Alam, Selangor Malaysia; 2 Sector for Biostatistic and Data Repository, National Institutes of Health Ministry of Health Shah Alam, Selangor Malaysia; 3 Institute for Health Behavioural Research, National Institutes of Health Ministry of Health Shah Alam, Selangor Malaysia; 4 Institute for Public Health, National Institutes of Health Ministry of Health Shah Alam, Selangor Malaysia; 5 Occupational Safety and Health Unit, National Institutes of Health Ministry of Health Shah Alam, Selangor Malaysia; 6 Faculty of Fisheries and Food Science, Universiti Malaysia Terengganu Terengganu Malaysia

**Keywords:** intermittent fasting, dry fasting, obesity, overweight, healthy plate

## Abstract

**Background:**

Overweight and obesity among adults are a growing global public health threat and an essential risk factor for various noncommunicable diseases. Although intermittent fasting is a generally new dietary approach to weight management that has been increasingly practiced worldwide, the effectiveness of 2 days per week dry fasting remains unclear.

**Objective:**

The Cardiometabolic and Anthropometric Outcomes of Intermittent Fasting study aims to determine the cardiometabolic, anthropometric, dietary intake, and quality of life changes among civil servants with overweight and obesity, following combined intermittent fasting and healthy plate (IFHP) and healthy plate (HP) and explore the participants’ experiences.

**Methods:**

We designed a mixed methods quasi-experimental study to evaluate the effectiveness of the IFHP and HP methods among adults with overweight and obesity. A total of 177 participants were recruited for this study, of which 91 (51.4%) were allocated to the IFHP group and 86 (48.6%) to the HP group. The intervention comprised 2 phases: supervised (12 weeks) and unsupervised (12 weeks). Data collection was conducted at baseline, after the supervised phase (week 12), and after the unsupervised phase (week 24). Serum and whole blood samples were collected from each participant for analysis. Data on sociodemographic factors, quality of life, physical activity, and dietary intake were also obtained using questionnaires during data collection.

**Results:**

Most of the participants were female (147/177, 83.1%) and Malay (141/177, 79.7%). The expected outcomes of this study are changes in body weight, body composition, quality of life, physical activity, dietary intake, and cardiometabolic parameters such as fasting blood glucose, 2-hour postprandial blood glucose, hemoglobin A1c, fasting insulin, and lipid profile.

**Conclusions:**

The Cardiometabolic and Anthropometric Outcomes of Intermittent Fasting study is a mixed methods study to evaluate the effectiveness of combined IFHP and HP interventions on cardiometabolic and anthropometric parameters and explore participants’ experiences throughout the study.

**Trial Registration:**

ClinicalTrials.gov NCT05034653; https://clinicaltrials.gov/ct2/show/NCT05034653

**International Registered Report Identifier (IRRID):**

RR1-10.2196/33801

## Introduction

### Background

The overweight and obesity epidemic has become one of the most alarming public health threats. Although largely preventable, the worldwide prevalence of obesity nearly tripled between 1975 and 2016 and shows an increasing trend. In 2016, >1.9 billion adults were overweight, and 650 million were obese. If this current trend continues, it is estimated that 2.7 billion and >1 billion adults will be overweight and obese, respectively, by 2025 [1]. In Malaysia, the National Health and Morbidity Survey (NHMS) 2019 reported that 50.1% of adults were either overweight (30.4%) or obese (19.7%), which increased compared with the NHMS 2011 (overweight 29.4% and obesity 15.1%) and 2015 (overweight 30% and obesity 17.7%) findings [2].

The relationship between obesity and poor health outcomes is well established. Despite the increased risk of noncommunicable diseases such as hypertension, diabetes, stroke, coronary heart disease, and certain cancers, a growing body of literature has demonstrated a positive relationship between obesity and various mental health issues such as depression and poor quality of life [3,4]. The World Health Organization has established multiple strategies that describe the actions that need to be taken by stakeholders at the global, regional, and local levels to combat obesity in adults and children. Furthermore, effective and feasible policy actions have been included in the “Global action plan on physical activity 2018-2030: more active people for a healthier world” to increase physical activity globally [1].

As obesity occurs because of a positive energy balance in the body, strategies for preventing and treating obesity mainly focus on dietary modification and increasing physical activity. A form of calorie restriction dietary protocol is intermittent fasting (IF), which encompasses various eating diet plans that cycle between fasting and nonfasting states over a defined period to create a negative energy balance, thereby inducing weight loss. Studies have shown that IF is effective in reducing body weight and improving metabolic outcomes [5,6]. Although the effects of wet IF have been well documented, the benefits of dry IF (except for Ramadhan fasting for Muslims) have not been clearly indicated in previous studies. Wet or water IF is defined as fasting during which all food and drink except water are restricted [7], whereas dry IF is complete fasting without any food and fluid intake [8]. In countries with predominantly Muslims, the practice of dry fasting during Ramadan and the voluntary 2 days per week fasting (Mondays and Thursdays) are widely conducted.

As portion size is a crucial determinant of energy intake, portion control by controlling serving size is another practical method for reducing calorie intake and promoting weight loss. This portion-control method has been widely practiced and studied worldwide, using different portion divisions depending on the culture and eating habits [9,10]. The Malaysia Healthy Plate, a portion-control dietary plan, was created to translate the messages in the Malaysia Dietary Guideline 2010 and Malaysia Food Pyramid 2010 [11]. It is a visual tool that emphasizes the quarter-quarter-half concept and provides a quick visual technique that helps ensure that the intake of food is within the recommended guidelines. Specifically, the Malaysia Healthy Plate is a single-meal guide that divides the plate into a quarter plate of grains or grain products; a quarter plate of fish, poultry, meat, or egg; and a half plate of fruits and vegetables [12].

### Objectives

Although weight loss has been reported in studies of conventional IF, the adaptive phenomenon of dry IF on 2 nonconsecutive days per week on cardiometabolic and anthropometric outcomes remain unclear. Similarly, although the Malaysian Healthy Plate policy has been widely practiced and publicized since 2010, the effectiveness of this eating plan in improving cardiometabolic risks and promoting weight loss is still not well documented. Thus, we established the Cardiometabolic and Anthropometric Outcomes of Intermittent Fasting study to determine the cardiometabolic, anthropometric, dietary intake, and quality of life changes among civil servants with overweight and obesity, following combined IF and healthy plate (IFHP) and healthy plate (HP) and explore the participants’ experiences throughout the study. We hypothesized that combining dry IF and HP diet protocols will improve these parameters more than HP alone.

## Methods

### Study Design

This is a quasi-experimental study applying a mixed study method that consists of 2 parts: quantitative and qualitative. The quantitative part involved allocating participants into 2 intervention arms: the combined IFHP group and the HP group and measuring the parameters of cardiometabolic risk and anthropometrics at baseline, month 3 and month 6. The qualitative part aimed to explore the facilitators and barriers that enabled or admonished the success of weight loss in the IFHP group through focus group discussions (FGDs) conducted at month 6.

### Study Site

The participants were allocated to each intervention arm according to the institutes in the National Institutes of Health in Setia Alam; the Institute for Medical Research, Jalan Pahang; and the Institut Latihan Kementerian Kesihatan Malaysia (Teknologi Makmal Perubatan), Jalan Pahang. The distance between Jalan Pahang and Setia Alam is approximately 40 km. The allocation was done in such a manner to avoid contamination bias. The study population had a relatively similar sociodemographic features, environment, facility, and nature of work. The allocation of the intervention was determined a priori and was based on the feasibility of monitoring the study participants.

### Study Population

#### Inclusion and Exclusion Criteria

Workers aged 19 to 59 years with a BMI of ≥23 kg/m^2^ (overweight or obese), ready to participate in the intervention (assessed through readiness to participate in screening), and providing informed consent were included in this study.

Workers who (1) had recent involvement in weight loss program or activity (IF, diet changes, or physical activity changes or any activities that were performed constantly to reduce weight); (2) were affected by any eating disorder; (3) were diagnosed with diabetes and hypertension (on medication) or other metabolic health disturbances such as thyroid disease, chronic kidney disease, malignancy, and polycystic ovarian syndrome; (4) were taking any medication or supplements that can affect study outcome; (5) were pregnant; and (6) had lack of capacity or language skills to independently follow the protocol were excluded from this study.

#### Sample Size

Sample size calculation was conducted using a power and sample size program. It followed the rules required for comparison between the 2 groups [13]. The sample size was estimated using the level of significance (α=.05) and power of the study (1-β=.80), minimum suggested difference (delta) of 5% (SD 10%) weight loss that may be achieved under this intervention, and the corresponding differences among groups. The assumption of 5% weight loss used in the sample size calculation was based on a review conducted by Ryan and Yockey [14], which stated that a minimum weight loss of 5% is needed to improve cardiometabolic risk such as hypertension, diabetes mellitus, and hyperlipidemia. The minimum sample size required for this study was 64 participants. Considering 40% attrition, the required sample size for each arm was 90 participants. A total of 180 participants were required for this study.

### Dietary Protocols

The combined IFHP regimen consisted of dry fasting from dawn to dusk for 2 days a week (Mondays and Thursdays) and practice of HP for the rest of the week. During the fasting days, the participants were encouraged to have a meal before dawn. No food or drink was allowed after dawn (approximately 13 hours) until sunset. They did not need to follow an HP diet on the fasting day. Smoking and sexual activity were also forbidden during the fasting day, following the Sunnah fasting obligation. Fasting adherence records were taken as 0, 1, or 2 fasting day or days per week. For the rest of the week, they were obligated to consume meals according to the HP concept. The female participants were discouraged from fasting during menstruation.

Participants in the HP group were asked to practice the HP concept daily: division of plate portions into a quarter for protein, a quarter for complex carbohydrates, and a half for fruits and vegetables. Participants were advised to practice HP for all 3 main meals per day. However, the practice of at least one HP meal per day is considered the minimum requirement for adherence to the dietary protocol. The research assistants (Nurul Hidayah binti Mat Yusoff and Norsyuhada binti Japri) monitored the intervention through a daily record of food intake picture (one meal per day) and a weekly fasting record.

### Study Phases

#### Recruitment Phase

The recruitment of participants for this study comprised two phases: (1) health screening and (2) readiness to participate in screening. An invitation to participate in the study was sent through Google Forms, emails, and phone calls. Volunteers were screened for inclusion and exclusion criteria by the study team. Those eligible were screened again for readiness to participate, which was conducted through face-to-face interviews. During the interviews, the participants’ readiness (motivation, willingness to commit, and enthusiasm) was assessed by a trained psychologist from the Institute for Health Behavioral Research. Only participants deemed ready to commit were included in the study. Informed consent, as well as a behavioral contract, was signed by each enrolled participant after being clearly explained the purpose of each document in the research. Both researchers and participants were unblinded to the intervention.

#### Intervention Phase

The overall duration of the intervention phase was 6 months, with 12 weeks in the supervised phase, followed by 12 weeks in the unsupervised phase. During the supervised phase, the participants started the diet protocol according to the designated intervention group. In the IFHP group, participants were reminded to fast, through a message sent to their mobile phones on the eve of fasting days, and accomplishments were recorded twice weekly. Participants in both groups were required to send a picture of one of their meals to the research assistant. In contrast, no fasting reminder, weekly fasting records, or meal pictures were sent during the latter unsupervised phase.

### Study Procedure

#### Quantitative Method

##### Data Collection

During the 6-month study duration, data collection was conducted at 3 points: baseline (before starting the supervised phase), month 3 (at the end of the supervised phase), and month 6 (at the end of the unsupervised phase). Participants were asked to answer questionnaires on social demography (during baseline only), the Food Frequency Questionnaire (FFQ), the International Physical Activity Questionnaire-Short Form (IPAQ-SF), and the Obesity and Weight-Loss Quality of Life (OWLQOL) Questionnaire. Anthropometry measurements and fasting blood samples were taken. Oral glucose tolerance test and body composition analysis were also performed.

##### Self-administered Questionnaire

The FFQ was used to determine the frequency of food and beverage consumption over the previous month. The questionnaire consisted of questions covering the frequency of cereals and cereal products, fast food, meat and meat products, fish and seafood, eggs, legumes and legume products, milk and milk products, vegetables, fruits, drinks, alcoholic drinks, confectionaries, bread spreads, and flavor intake. The validated Malay version of the FFQ used in this study consisted of 165 items, and the participants required approximately 30 minutes to answer the questions at each point of data collection [15]. The records were analyzed using Nutritionist Pro Nutrition Analysis Software 7.8.0 (Axxya Systems, 2021) to determine their energy and macronutrient intake.

To measure the quality of life, a validated Malay version of the OWLQOL questionnaire was used. The OWLQOL is a self-administered questionnaire that assesses participants’ feelings about obesity and their efforts in weight loss [16]. The 17 OWLQOL items consist of 7-point scale responses ranging from 0 (*not at all*) to 6 (*a very great deal)*. The score for each item was reversed before the total score was obtained. Consequently, it was transformed to a scale of 0 to 100, with a higher score indicating better quality of life [17]. The Malay version of the questionnaire has been validated among 28 female health staff with overweight and obesity, with a Cronbach *α* of .953 [18]. The participants needed approximately 10 minutes to complete the questionnaire.

The IPAQ-SF was used to measure the participants’ physical activity in the past week. The questionnaire was validated for use by adults in 12 countries [19]. In this study, we used the Malay version of the IPAQ-SF, which was validated in a study using data obtained from the NHMS 2011 [20]. The participants were requested to record how many days in the past week they spent on specific activities (vigorous and moderate activities and walking) for at least 10 minutes and the amount of time (in minutes) they engaged in a particular activity on an ordinary day. Physical activity level was calculated as the energy expenditure or metabolic equivalent task (MET) minutes per week (MET-minutes per week) based on the IPAQ scoring protocol [21]. To obtain MET scores for each activity, the total minutes spent on vigorous activity, moderate-intensity activity, and walking over the last 7 days were multiplied by 8.0, 4.0, and 3.3, respectively. The total physical activity score was calculated as the sum of all the MET scores from the 3 activity groups. Physical activity can also be categorized into low, moderate, and high physical activity levels, based on the scoring protocol available in the IPAQ website guidelines [21].

Sedentary behavior was also measured in this study. The question used to measure sedentary behavior was included in the IPAQ-SF questionnaire, based on the IPAQ sitting question. Participants were asked to state the total time they spent (hours) sitting or lying down, whether in the workplace, at home, or while traveling, excluding the time spent sleeping, on a typical day. The total daily sitting time was used as an indicator of sedentary behavior.

##### Anthropometric Measurements

Body weight and height were measured using a seca electronic column scale (SECA GmbH and Co KG) in kilograms and centimeters to the nearest 0.1 kg and 0.1 cm, respectively. Body weight was measured in light clothing, and participants were asked to remove their outer garments and shoes. BMI was calculated by dividing weight by height squared (kg/m^2^). As only participants with overweight and obesity were included, we categorized them into overweight (23.0-27.4 kg/m^2^), preobese (27.5-32.4 kg/m^2^), obese class I (32.5-37.4 kg/m^2^), and obese class II (≥37.5 kg/m^2^), based on the cutoff points for public health action for Malaysia [22].

Waist and hip circumferences were measured using a SECA measuring tape (SECA), to the nearest 0.1 cm with the participant standing. Waist circumference was measured at the midpoint between the top of the iliac crest and the lower margin of the last palpable rib, whereas hip circumference was measured at the widest diameter around the buttocks. The waist-to-hip ratio was calculated by dividing the waist measurement by the hip measurement. On the basis of the World Health Organization cutoff points, waist-to-hip ratio of ≥0.90 cm (men) and ≥0.85 cm (women) are abnormal, and the risks of metabolic complications substantially increased beyond these points [23].

Blood pressure was measured using an automated upper arm device (Omron Automated Blood Pressure Monitor; HEM 7130). Body composition parameters such as fat mass and fat-free mass were measured using a tetrapolar bioimpedance multifrequency InBody 770 analyzer (Biospace). Personal profiles (age, height, weight, and sex) were entered upon measurement reading.

For each parameter measured 2 measurements were taken, and the average of the 2 measurements was calculated to minimize the measurement error.

##### Biochemical Testing

Before blood collection, all participants were required to fast overnight for approximately 8 to 10 hours. Approximately 15 ml of fasting venous blood was taken from participants by the medical officers for standard biochemical tests such as fasting blood glucose, hemoglobin A_1c_, fasting insulin, and fasting lipid profile such as triglycerides, total cholesterol, high-density lipoprotein cholesterol, and low-density lipoprotein (LDL) cholesterol. The oral glucose tolerance test was performed by asking the participant to drink a 250-mL solution that consists of 75 g of glucose, and another 5 mL of venous blood was collected 2 hours after the oral glucose tolerance test.

Blood samples were processed within 2 hours, and aliquots of serum or plasma samples were stored at −20 °C before analysis. Excess blood samples will be stored for up to 20 years and will be used for future research, as clearly stated in the consent form. The hemoglobin A_1c_ level was determined by cationic exchanged high-performance liquid chromatography (Adams A_1c_ HA-8160; Arkray Inc) following the National Glycohemoglobin Standardization Programme Guidelines.

Fasting plasma glucose, triglycerides, total cholesterol, high-density lipoprotein cholesterol, and LDL cholesterol were analyzed using an automated analyzer (Dirui CS-400) with reagents purchased from Randox Laboratories. Consent was obtained from the participant for permission to extract DNA or RNA and store the remaining samples at −80 °C for future biomarker research related to obesity.

##### DNA Extraction

Genomic DNA was isolated from frozen peripheral blood samples using the QIAamp Blood Mini Extraction Kit, according to the manufacturer’s protocol (Qiagen). Briefly, 20 µL QIAGEN Protease will be added in 200 µL of blood sample, followed by 200 µL Buffer AL. The mixture was vortexed thoroughly and incubated at 56 °C for 10 minutes. Later, 200 µL of absolute ethanol will be added to the mixture before being transferred to a QIAamp Mini spin column and centrifuged at 8000 rpm. Next, 2 washing steps will be performed using washing buffers AW1 and AW2. Finally, 100 µL of distilled water will be added and incubated at room temperature for 1 minute followed by centrifuging at 8000 rpm for 1 minute to elute the DNA. The quality and quantity of the extracted DNA will be quantified using NanoDrop before being stored at −20 °C for future use.

#### Qualitative Method

The FGDs were conducted after month 6 of the study to explore the experience the participant went through, including the enablers and barriers that led to their weight loss outcomes, and obtain their insights on how to improve the intervention.

A trained psychologist from the Institute for Behavioral Research conducted the FGDs using a predetermined outlined interview guide with probes. An audio recorder was used to record conversations and for transcription. Each FGD took approximately 60 to 90 minutes to complete. In total, 4 groups were involved in the FGD, in which 2 groups consisted of participants who successfully reduced weight by at least 4% from their baseline weight, whereas the other 2 groups were among those who did not meet the requirements for weight loss as predetermined by the study parameters.

A summary of the study outcomes, based on part of the study, is listed in Table 1.

**Table 1:** Summary of measurements undertaken within Cardiometabolic and Anthropometric Outcomes of Intermittent Fasting study and their associated outcome variables.

Measurements^a^		Time points				Instrument		Number of measures at each time point		Outcome variables
		Baseline	Month 3	Month 6						
**Quantitative part**										
	Demographic data	Yes	No	No	Questionnaire		Once		Sex Ethnicity Age Highest education status Job category Monthly salary Smoking history Background illness	
	Weight	Yes	Yes	Yes	SECA Electronic Column Scale (SECA GmbH and Co KG)		Twice in kg, to the nearest 0.1 kg		BMI in kg/m2, categorized based on Asian population standard	
	Height	Yes	Yes	Yes	SECA Electronic Column Scale		Twice in cm, to the nearest 0.1 cm		BMI in kg/m2, categorized based on Asian population standard	
	Waist circumference	Yes	Yes	Yes	SECA measuring tape (SECA)		Twice in cm, to the nearest 0.1 cm		Waist-to-hip ratio based on the World Health Organization cutoff points	
	Hip circumference	Yes	Yes	Yes	SECA measuring tape		Twice in cm, to the nearest 0.1 cm		Waist-to-hip ratio based on the World Health Organization cutoff points	
	Blood pressure	Yes	Yes	Yes	An automated upper arm device (Omron Automated Blood Pressure Monitor; HEM 7130)		Twice (third measure if error reading or if one value outside normal range)		Systolic and diastolic blood pressure in mm Hg	
	Body composition	Yes	Yes	Yes	InBody 770 analyzer (Biospace)		Once		Body fat % Body fat mass, kg Skeletal muscle mass, kg	
	Quality of life	Yes	Yes	Yes	Obesity and Weight-Loss Quality of Life Questionnaire		Once		Self-reported quality of life in the past month	
	Physical activity	Yes	Yes	Yes	International Physical Activity Questionnaire-Short Form		Once		Self-reported physical activity for the past week, in metabolic equivalent task minutes per week	
	Dietary intake	Yes	Yes	Yes	Food Frequency Questionnaire		Once		Self-reported dietary intake for the past month	
	Biochemical testing	Yes	Yes	Yes	Liquid chromatography (Adams A_1c_ HA-8160; Arkray Inc); automated analyzer (Dirui CS-400)		Once		Fasting blood glucose 2-hour postprandial blood glucose Hemoglobin A1c Fasting lipid profile^b^ Fasting insulin	
**Qualitative part**										
	Readiness to participate in screening	Yes	No	No	Behavioral contract and informed consent		Once, during recruitment phase		N/A^c^	
	Focus group discussion	No	No	Yes	Predetermined outlined interview guide with probes; an audio recorder		Once, after unsupervised intervention phase at the end of month 6		Themes generated from codes from each category	

^a^Measurements were carried out by trained research staff using standard protocols.

^b^Fasting lipid profile parameters: total cholesterol, triglycerides, low-density lipoprotein cholesterol, and high-density lipoprotein cholesterol.

^c^N/A: not applicable.

### Training of Study Team

Before the beginning of data collection, a 3-day workshop was conducted to train the research team members on the skills needed during data collection. The training included techniques for anthropometric measurements, body composition measurements using the InBody 770 analyzer, and explanation on questionnaires used in the study. Presentation on data collection workflow, intervention supervision processes, and biochemical testing procedures was also included in the training. In addition, selected study members were trained by a psychologist from the Institute for Behavioral Research on conducting readiness to participate in screening during the recruitment phase.

### Data Management

To ascertain that data collection and record keeping are conducted efficiently, a data collection booklet was developed and assigned to each participant in this study. This booklet consisted of 5 sections (sociodemographic, quality of life, physical activity, dietary record, and anthropometry measurements) sorted into 3 parts representing each point of data collection: baseline, month 3, and month 6. This booklet was used as a data-collection tool to record the responses and measurements of the participants.

The data recorded in the booklet were entered into a database at the end of the study. The data cleaning procedure was conducted by crosschecking all the entered data in the database and booklet and exploring the data to detect any significant outliers that possibly resulted from measurement errors or data entry.

Data for qualitative part were collected using an audio recorder, and each recording was transcribed verbatim by the qualitative team. Each transcript was checked by an independent member and confirmed by a transcriber and interviewer. Consent for audio recording was obtained before the interviews.

### Data Analysis

Analysis was conducted using the SPSS software (version 25; IBM Corp). Data for continuous variables were presented as mean (SD) or as median (IQR) for nonnormally distributed data. For categorical variables, frequencies were calculated and are presented as percentages. Variables were compared using the independent 2-tailed *t* test or Mann-Whitney *U* test for continuous variables and the chi-square or Fisher exact (n≤5 in any cell) test for categorical variables. All statistical tests were 2-sided, and the significance level was set at *P*<.05. In further analysis, repeated measures ANOVA will be used to compare the within- and between-group changes in the outcomes, adjusted for possible confounders such as age, ethnicity, and gender.

Data from the FGDs were analyzed using thematic analysis. Interviews transcribed verbatim were independently read by a qualitative researcher (MZJ) to identify the preliminary codes. An interpretivist approach was taken to interpret and code participants’ experiences for the entire study. In this way, the coding of participants’ feedback was performed without assumptions or subjective interpretation by the researchers. Meaning units were reviewed, identified, and sorted into codes, before grouping them into categories. Finally, through consensus, the content of each category group was summarized and grouped into main themes. The best representative participants’ quotes for each theme were chosen to support the results. Thematic analysis was used, where initial open codes were generated from the data, after which the codes were organized into larger themes.

### Ethics Approval and Informed Consent

Ethics approval for this study has been obtained from the Medical Research & Ethics Committee, Ministry of Health Malaysia (NMRR-19-3261-51726). Before recruitment, written informed consent was obtained from each participant, including the storage of samples for biochemical and future DNA analyses. Participants were also informed that future research will be related to medical conditions and current interventions and that their privacy will be protected. Before enrollment, all participants were fully informed of the potential risks associated with engaging in this study. They were free to withdraw from the study at any time during their participation. This study was conducted in full conformity with the current revision of the Declaration of Helsinki and International Council for Harmonisation Guidelines for Good Clinical Practice.

## Results

### Study Participants

**Figure 1:**
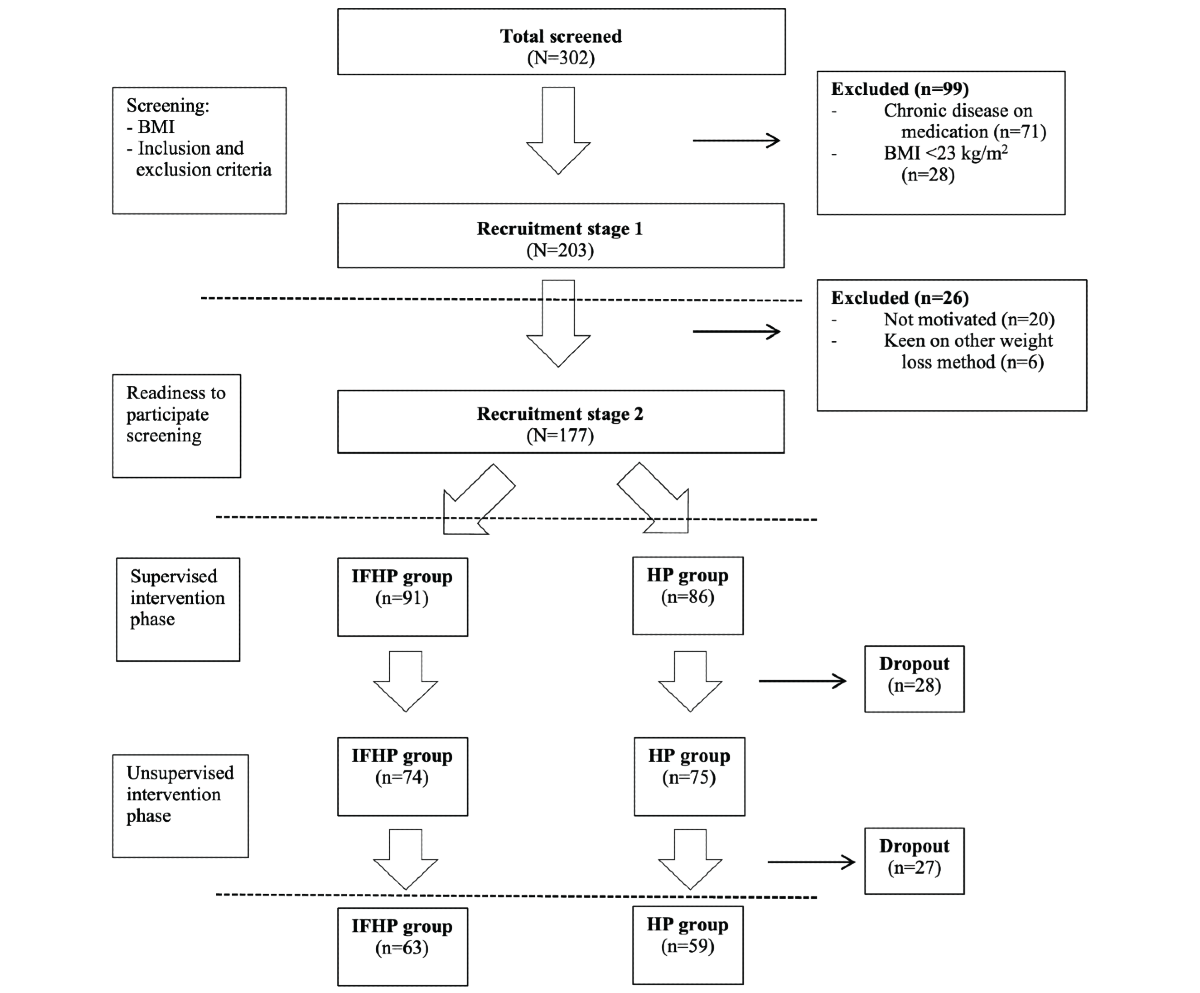
Flow chart of number of study participants throughout the study. HP: healthy plate; IFHP: intermittent fasting and healthy plate.

A total of 302 volunteers were interested in joining the study and underwent the screening process. After screening for BMI and eligibility during the first stage of recruitment, of 302 volunteers, only 203 (67.2%) were found eligible. Most volunteers were excluded owing to a BMI of <23 kg/m^2^ or having chronic diseases on medication, such as diabetes mellitus and hypertension. During the readiness to participate in screening (stage 2 recruitment phase), 27 volunteers were excluded for various reasons but were not limited to reasons such as commitment issues, furthering education, or moving away. Hence, there were a total of 177 participants recruited in this study; 91 (51.4%) in the IFHP group and 86 (48.6%) in the HP group (Figure 1). During the supervised intervention period, 28 participants withdrew from our study in both groups (IFHP: 16/28, 57%; HP: 12/28, 43%), whereas 27 withdrew during the unsupervised period (IFHP: 12/27, 44%; HP: 15/27, 56%). The reasons for withdrawal included pregnancy (13/55, 24%), inability to commit to study intervention (24/55, 44%), transfer to a different workplace (8/55, 15%), being diagnosed with hypertension, diabetes, hypercholesterolemia on medication (5/55, 9%), and other reasons (5/55, 9%). There were 63 and 59 participants who completed the study in the IFHP and HP groups, respectively.

### Sociodemographic Characteristics

Table 2 shows the sociodemographic characteristics of the study participants according to the intervention groups. Most of the participants were female (147/177, 83.1%) and had a diploma or degree with the highest education status (121/177, 68.4%). The mean age of the participants was 34.47 (SD 7.40) years. In terms of ethnicity, 79.7% (141/177) of the participants were Malay, followed by other races (20/177, 11.3%), Indian (12/177, 6.8%), and Chinese (4/177, 2.3%). Furthermore, most participants from the groups reported a monthly household income above Malaysian Ringgit 6000 (US $1422; IFHP: 35/91, 39%; HP: 41/86, 48%). Approximately 90.4% (160/177) of the participants in each group denied any history of smoking (IFHP: 82/91, 90%; HP: 80/86, 93%); of the 15 participants who had a history of smoking, only 4 (27%) participants from each group were current smokers (IFHP: 4/9, 44%; HP: 4/6, 67%). Regarding background illness history, only 5.1% (9/177) of participants reported being diagnosed with hyperlipidemia, whereas 1.1% (2/177) of participants were diagnosed with hypertension. However, all patients denied taking any medication for the illness (Table 2).

A comparison of anthropometric measurements of the study participants among the intervention groups is presented in Table 3. On the basis of the BMI category, most participants were preobese (69/177, 38.9%), followed by overweight (63/177, 35.6%), obese class I (32/177, 18.1%), and obese class II (13/177, 7.3%). Although the BMI of the participants in the IFHP group was slightly higher than that of the participants in the HP group, the difference was not statistically significant (*P*=.13; Table 3). Overall, no significant differences were observed in sociodemographic characteristics and anthropometric measurements between the 2 groups at baseline, except for ethnicity and job category (*P*<.05; Tables 2 and 3).

After 6 months after the intervention, 21 participants were interviewed for their feedback across 4 different groups: 2 being successful in their weight loss attempts and the other 2 groups that did not meet the requirements of weight loss reduction. Four main themes were constructed from the feedback given: efficacy toward the intervention, barriers and facilitators that enable or admonish weight loss attempts, support during the intervention, and perceived sustainability of the intervention. This feedback serves as a platform for researchers to improve their future interventions.

**Table 2:** Sociodemographic characteristics of participants in both intervention groups (N=177).

Characteristics					Participants			Intervention groups						*P* value^a^	
								Intermittent fasting and healthy plate (n=91)			Healthy plate (n=86)				
**Sex, n (%)**															.32^b^
	Male			30 (16.9)			18 (19.8)			12 (14)					
	Female			147 (83.1)			73 (80.2)			74 (86)					
**Ethnicity, n (%)**															.02^c^
	Malay			141 (79.6)			80 (87.9)			61 (70.9)					
	Chinese			4 (2.3)			0 (0)			4 (4.7)					
	Indian			12 (6.8)			4 (4.4)			8 (9.3)					
	Others			20 (11.3)			7 (7.7)			13 (15.1)					
Age (years), mean (SD)					34.47 (7.40)			33.82 (7.50)			35.15 (7.27)			.23^d^	
**Highest education status, n (%)**															.95^b^
	Secondary school			24 (13.6)			13 (14.3)			11 (12.8)					
	Diploma or degree			121 (68.4)			62 (68.1)			59 (68.6)					
	Master or PhD			32 (18.1)			16 (17.6)			16 (18.6)					
**Job category, n (%)**															<.001^c^
	Research officer			14 (7.9)			7 (7.7)			7 (8.1)					
	Medical officer			8 (4.5)			5 (5.5)			3 (3.5)					
	Science officer			5 (2.8)			3 (3.3)			2 (2.3)					
	Medical laboratory technologist			50 (28.2)			16 (17.6)			34 (39.5)					
	Assistant Research Officer			8 (4.5)			6 (6.6)			2 (2.3)					
	Clerk			26 (14.7)			23 (25.3)			3 (3.5)					
	Others			66 (37.3)			31 (34.1)			35 (40.7)					
**Monthly household income (Malaysian Ringgit), n (%)**															.63^c^
	<1500 (US $357)			3 (1.7)			2 (2.2)			1 (1.2)					
	1500 to <3000 (US $357 to <US $711)			34 (19.2)			20 (22.0)			14 (16.3)					
	3000 to <4500 (US $711 to <US $1067)			37 (20.9)			18 (19.8)			19 (22.1)					
	4500 to <6000 (US $1067 to <US $1422)			27 (15.3)			16 (17.6)			11 (12.8)					
	≥6000 (≥US $1422)			76 (42.9)			35 (38.5)			41 (47.7)					
**Smoking history, n (%)**															.34^b^
	**Ever smoked**			15 (8.5)			9 (9.9)			6 (7)					
		Current smoker	8 (53.3)			4 (44.4)			4 (66.7)						
		Ex-smoker	7 (46.7)			5 (55.6)			2 (33.3)						
	Never smoked			162 (91.5)			82 (90.1)			80 (93)					
**Background illness, n (%)**															
	Hypertension			2 (1.1)			1 (1.1)			1 (1.1)			.74^c^		
	Hyperlipidemia			9 (5.1)			6 (6.6)			3 (3.5)			.498^c^		
	Heart disease			1 (0.6)			0 (0)			1 (1.2)			.49^c^		
	Others			1 (0.6)			0 (0)			1 (1.2)			.49^c^		

^a^*P* values of <.05 are considered statistically significant.

^b^*P* values derived from Pearson chi-square test.

^c^*P* values derived from Fisher exact test.

^d^*P* values derived from independent 2-tailed *t* test.

**Table 3:** Anthropometric measurements of participants in both intervention groups (N=177).

Anthropometric measurements		Value	Intervention groups		*P* value^a^	
			Intermittent fasting and healthy plate (n=91)	Healthy plate (n=86)		
Systolic blood pressure (mm Hg), mean (SD)		115 (13.13)	116 (12.13)	114 (14.07)	.22^b^	
Diastolic blood pressure (mm Hg), mean (SD)		80 (9.43)	80 (9.45)	79 (9.44)	.52^b^	
Heart rate (beats per minute), mean (SD)		79 (10.48)	78 (8.51)	80 (12.24)	.36^b^	
Waist circumference^e^ (cm), mean (SD)		92.46 (10.88)	93.49 (10.70)	91.36 (11.03)	.20^b^	
Hip circumference^e^ (cm), mean (SD)		108.86 (8.66)	109.39 (9.02)	108.31 (8.29)	.41^b^	
Weight (kg), median (IQR)		71.70 (18.70)	72.50 (18.55)	70.60 (16.28)	.13^c^	
Height (cm), mean (SD)		158.36 (7.09)	158.67 (7.13)	158.04 (7.08)	.56^b^	
BMI (kg/m^2^), median (IQR)		28.43 (6.50)	28.48 (7.25)	28.39 (5.70)	.18^c^	
**BMI category, n (%)**						.51^d^
	Overweight	63 (35.6)	32 (35.2)	31 (36)		
	Preobese	69 (39)	32 (35.2)	37 (43)		
	Obese class I	32 (18.1)	20 (22)	12 (14)		
	Obese class II	13 (7.3)	7 (7.6)	6 (7)		
Waist-to-hip ratio^e^, mean (SD)		0.85 (0.06)	0.85 (0.06)	0.84 (0.06)	.20^b^	

^a^*P* values of <.05 are considered statistically significant.

^b^*P* values derived from independent 2-tailed *t* test.

^c^*P* values derived from the Mann-Whitney *U* test (nonnormally distributed data).

^d^*P* values derived from Pearson chi-square test.

^e^Missing data: waist circumference (n=1), hip circumference (n=1), waist-to-hip ratio (n=1).

## Discussion

### Principal Findings

We hypothesized that there would be a significant improvement in cardiometabolic and anthropometric parameters among participants in the IFHP group following the intervention after 3 and 6 months. We expected that these changes would also be present among participants in the HP group, but the degree of change would be more prominent among participants in the IFHP group. We also believe that the effectiveness of the intervention in improving cardiometabolic and anthropometric outcomes was driven by both personal motivation and a strong support system.

The preliminary results showed no significant differences in most sociodemographic characteristics and anthropometric measurements of the participants between the 2 intervention groups. The significant difference observed in job categories is most likely because of the departmental units stationed at the Institute for Medical Research Jalan Pahang being diagnostic based; thus, there was a higher proportion of medical laboratory technologists in the HP group than in the IFHP group.

### Comparison With Prior Work

On the basis of a meta-analysis by Harris et al [24], there was no significant difference in weight loss between continuous and intermittent energy restriction [24]. Thus, it can be concluded that to reduce body weight, intermittent energy restriction is an alternative method to continuous energy restriction and may be preferred because of its feasibility and practicality.

Despite robust evidence supporting the effectiveness of wet IF in reducing weight and improving cardiometabolic risks, the data on dry IF, especially 2 days per week fasting (such as fasting on Mondays and Thursdays) remain limited. Voluntary Sunnah fasting on Monday and Thursday is widely practiced by Muslims worldwide and is culturally more acceptable in Malaysia, as most Malaysians are Muslims. In 2013, Teng et al [25] compared the effect of dry fasting on Mondays and Thursdays combined with calorie restriction (fasting calorie restriction), with the control of metabolic parameters. They found that participants in the fasting calorie restriction groups showed significant interaction effects on body weight, BMI, body composition, blood pressure, total cholesterol, and LDL cholesterol compared with the participants in the control group [25]. However, as fasting was combined with calorie restriction and compared with the control group, the sole effect of fasting was not sought. Furthermore, the study was conducted among Malay men aged 50 to 70 years, thus limiting the generalizability of the findings to the general population. Meanwhile, this study compared the combined IFHP with the use of HP method alone and involved adults (aged >18 years) of both sexes.

### Strengths and Limitations

A strength of our study is that we applied both quantitative and qualitative methods. The mixed method allows us to measure the effectiveness of the intervention and explore the challenges of practicing it simultaneously. This integrated framework is crucial for a better understanding of the challenges faced during dietary intervention in obesity prevention programs so that it can be improvised in future programs to improve the outcomes and sustainability of health changes.

The main limitation of our study is the effect of the movement control order due to the COVID-19 pandemic on intervention compliance and weight management. The implementation of movement control orders and work from home for those working would limit their physical activity, expose them to unhealthy eating, reduce their motivation toward weight loss, and affect their control of food intake. Studies have shown that social lockdowns have negative consequences on weight-related behavior and weight management [26,27].

### Future Directions

Adherence to dietary interventions is essential to ensure the validity of research findings and, most importantly, to ascertain the sustainability of the desired outcomes beyond the research period. As described by the World Health Organization, adherence is “the extent to which a person’s behavior-taking medication, following a diet, and executing lifestyle changes, corresponds with agreed recommendations from a health care provider” [28]. In this study, we examined the elements of adherence in both quantitative and qualitative parts. Quantitatively, we investigated adherence to intervention and sustainability of outcomes’ changes in the unsupervised intervention phase. We measured the outcomes at the end of the phase and compared them with those of the previous 2 parameters. In the qualitative part, we assessed readiness to participate in screening during the recruitment phase, in which the motivation and readiness to comply with intervention protocols were assessed. In addition, FGDs were conducted at the end of the study to explore the participants’ experiences while undergoing the intervention, including barriers and enablers that influence their adherence to the diet protocols.

We plan to disseminate the study results to collaborating institutes and organizations, study participants, and respective stakeholders. These findings will be submitted to 2 peer-reviewed journals and presented at academic conferences.

### Conclusions

With the increase in the prevalence of overweight and obesity worldwide, a weight loss method that is not only effective but also practical and easy to comply with is required. Although IF has been widely practiced and studied, data on the effectiveness of dry fasting in reducing weight and cardiometabolic risk are limited. We established the Cardiometabolic and Anthropometric Outcomes of Intermittent Fasting study to determine the effectiveness of a combined IFHP and HP in improving anthropometric and cardiometabolic outcomes among civil servants with overweight and obesity. The mixed methods study was designed to measure the changes quantitatively, reflect the participants’ points of view, and ensure that the study findings are grounded in their experiences. The study findings and their in-depth explanation may contribute to the development of more effective and feasible obesity prevention methods, improve current health policies, and provide new insights that will stimulate new research questions in the future.

## Abbreviations

FFQ: Food Frequency Questionnaire
FGD: focus group discussion
HP: healthy plate
IF: intermittent fasting
IFHP: intermittent fasting and healthy plate
IPAQ-SF: International Physical Activity Questionnaire-Short Form
LDL: low-density lipoprotein
MET: metabolic equivalent task
NHMS: National Health and Morbidity Survey
OWLQOL: Obesity and Weight-Loss Quality of Life
